# Strengthening Care Delivery in Primary Care Facilities: Perspectives of Facility Managers on the Immunization Program in Kenya

**DOI:** 10.15171/ijhpm.2018.83

**Published:** 2018-09-06

**Authors:** Rose N. Chesoli, Roseanne C. Schuster, Stephen Okelo, Moshood O. Omotayo

**Affiliations:** ^1^University of Nairobi, Nairobi, Kenya.; ^2^Center for Global Health, Arizona State University, Tempe, AZ, USA.; ^3^State University of New York at Buffalo, Buffalo, NY, USA.; ^4^Massachusetts General Hospital, Boston, MA, USA.; ^5^Harvard Medical School, Boston, MA, USA.

**Keywords:** Kenya, Immunization, Primary Healthcare, Child Health, Health Systems

## Abstract

**Background:** Primary healthcare facility managers (PHFMs) occupy a unique position in the primary healthcare system, as the only cadre combining frontline clinical activities with managerial responsibilities. Often serving as ‘street-level bureaucrats,’ their perspectives can provide contextually relevant information about interventions for strengthening primary healthcare delivery, yet such perspectives are under-represented in the literature on primary healthcare strengthening. Our objective in this study was to explore perspectives of PHFMs in western Kenya regarding how to leverage human resource factors to improve immunization programs, in order to draw lessons for strengthening of primary healthcare delivery.

**Methods:** We employed a sequential mixed methods approach. We conducted in-depth interviews with key informants in Kakamega County. Emergent themes guided questionnaire development for a cross-sectional survey. We randomly selected 94 facility managers for the survey which included questions about workload, effects of workload on immunization program, and appropriate measures to address workload effects. Participants provided self-assessment of their general motivation at work, their specific motivation to ensure that all children in their catchment areas were fully immunized, and recommendations to improve motivation. Participants were asked about frequency of supervisory visits, supervisor activities during those visits, and how to improve supervision.

**Results:** The most frequently reported consequences of high workload were reduced accuracy of vaccination records (47%) and poor client counseling (47%). Hiring more clinical staff was identified as an effective remedy to high workload (69%). Few respondents (20%) felt highly motivated to ensure full immunization coverage and only 13% reported being very motivated to execute their role as a health worker generally. Increasing frequency of supervisory visits and acting on the feedback received during those visits were mostly perceived as important measures to improve program effectiveness.

**Conclusion:** Besides increasing the number of staff providing clinical care, PHFMs endorsed introducing some financial incentives contingent on specified targets and making supervisory visits meaningful with action on feedback as strategies to increase program effectiveness in primary healthcare facilities in Kenya. Targeting health worker motivation and promoting supportive supervision may reduce missed opportunities and poor client counseling in primary healthcare facilities in Kenya.

## Background

Primary healthcare facilities are often the most accessible point of care in the public healthcare system for communities in sub-Saharan Africa. Effective implementation of programs in these facilities depend on careful planning and coordination. Primary healthcare facility managers (PHFMs), commonly known as the ‘facility in-charge’ in Kenya, often play this role. PHFMs are clinical service providers who are tasked with management of the facility where they work, typically the only facility role to combine clinical and management responsibilities in the primary healthcare system. PHFMs often exercise discretion in policy implementation, adapting programs to their context and giving expression to policy and guidelines in the communities they serve.^1^ They serve as “street-level bureaucrats,” and in effect define and shape how national and regional health policies and initiatives are experienced by the communities they serve.^2^

Effective delivery of public health programs depends on a skilled and motivated workforce.^3,4^ The size and distribution of the health workforce is crucial in intervention delivery.^5^ However, the effectiveness of primary healthcare programs, including immunization, also depends on motivation and productivity.^6^ In addition to the heavy workload associated with an under-resourced workforce, low motivation and key workplace environmental factors such as inadequate supportive supervision negatively impact service delivery in low and middle-income countries.^7^ This human resource crisis is co-occurring with a high infectious disease burden and need for immunization, as well as a burgeoning chronic disease burden among other demands on the healthcare system.^8^ Thus, human resource factors have been identified as potential targets for strengthening primary healthcare services, including immunization services.^9,10^

Analyzing PHFMs perspectives on how to leverage human resource factors to improve immunization programming provides a window into understanding primary healthcare strengthening. Immunization is one of the most cost-effective public health interventions for preventing child morbidity, mortality, and life time disabilities.^11,12^ Globally, immunization prevents more than 2.5 million child deaths each year, but over 19 million infants are left unprotected against vaccine-preventable diseases (VPDs), with the poorest effective coverage in sub-Saharan Africa.^13^ Despite recent increase in vaccine coverage globally, optimal levels for herd immunity have not been achieved. Vaccine preventable diseases, particularly pneumonia and diarrhea, remain leading causes of child morbidity and mortality in low- and middle-income countries.^14^ In Kenya for instance, VPDs are high contributors to child mortality and the diarrhea-tetanus-pertussis vaccine (DTP3) coverage estimate is 78%.^15^

Advancements in immunology and vaccine development mean that as new vaccines are added to an already complex immunization schedule,^16,17^ even more efficient and organized management systems are needed to ensure appropriate implementation and coverage.^18^ Addition of new vaccines creates more pressure on already burdened primary healthcare providers who have to integrate timely delivery of existing vaccines with learning protocols of newly introduced vaccines as well as other clinical and administrative responsibilities.^19^

PFHMs may face both demand- and supply-related factors in increasing routine vaccination coverage. Inadequate coverage is often driven by patients’ geographical distance to health facilities, poverty, and lack of trust in the healthcare system.^7,20^ However, supply related factors are also key determinants of coverage, including vaccine availability, vaccine infrastructure and storage, and human resources.^21^ A recent qualitative analysis of perspectives of government officials on internal accountability in Nigeria’s routine immunization programs highlighted the importance of human resource factors and workplace environment for program performance.^22^

In Kenya, PHFMs have broad responsibilities related to the immunization program, ranging from forecasting vaccine needs and data management, acquisition of vaccine stock from regional depots, maintenance of stock in the facility, management of antenatal and child care clinics, and coordination of community healthcare workers.^23^ Despite their unique position, there is paucity of studies documenting PHFM perspectives on how to improve program delivery, or their insights into challenges and strategies for last mile delivery.

Therefore, our objective in this paper is to explore perspectives of PHFMs in western Kenya regarding the influence of human resource factors on immunization programs in order to draw lessons for strengthening of primary healthcare delivery.

## Methods

### Study Setting

The study was carried out in Kakamega County in western Kenya. Kakamega County is the largest rural county and second most populous county in Kenya, after Nairobi. It is one of the counties with low immunization coverage rates at 62.2%, and relatively high under-five mortality rate of 90 deaths/1000 live births.^24^ The healthcare delivery system of the county primarily comprises government and faith-based primary healthcare facilities and a few private healthcare facilities. At the time of this study, there was one county referral hospital, 11 sub-county hospitals, 40 health centers and 96 dispensaries, all providing immunization services. The central vaccine depot for western Kenya was located in Kakamega town. Coordination of the immunization program was anchored at the county health management team, but the sub-county health management team also had the latitude of engaging with the regional office directly.

### Study Design

This study used mixed sequential methods design, involving two phases: in-depth interviews with key informants, followed by cross-sectional surveys with PHFMs. Data collection took place between January 2015 and June 2015. In this report, we present the findings from the survey only.

### Survey Development: Formative, In-Depth Interviews

Methods for the in-depth interviews have been described in detail elsewhere.^25^ Briefly, in-depth interviews were conducted with 14 key informants who were primarily members of the county and sub-county health management teams. Key informants were purposively sampled to maximize geographic diversity (eg, rural and urban), experience with maternal immunization, experience with childhood immunization, and professional cadre. Open ended questions were asked of the interviewees, with specific probes. Respondents were asked about their perceptions of problems with the delivery of immunization programs at primary healthcare facilities, and were probed specifically about infrastructure, human resources, data use, and community healthcare seeking behavior. Transcripts were analysed using the constant comparative method.^26^ Emergent themes from the interviews were used to guide questionnaire development for the cross-sectional survey.

### Survey Instrument

The survey questions were developed based on themes identified from the in-depth interviews. Major themes were used as topical headings for the cross-sectional survey questionnaire, and sub-themes were used to develop specific questions and response options. The survey included questions about factors contributing to PHFM workload, the effects of workload on immunization program delivery in their facility, and appropriate measures to reduce workload effects. We asked participants to provide self-assessment of their general motivation at work, their specific motivation to ensure that all children in their catchment areas were adequately immunized, and which measures they recommend to improve staff motivation. The final section included questions on the frequency of supervisory visits in the last three months, supervisor activities during those visits, and suggestions to improve supervisory visits. Each question stem had multiple answer options that were not mutually exclusive and respondents were requested to answer yes or no to each answer option, except for questions on self-rating of motivation and frequency of supervisory visits, which had mutually exclusive answer options from which respondents were requested to choose one option. The instrument was reviewed by an immunization expert in Kenya and a social science and health systems researcher for content validity. It was also pretested with healthcare providers in the neighboring Vihiga County in western Kenya and revised based on feedback from the reviews and pretesting.

### Sampling Strategy and Data Analysis

At the time of survey, there were 125 primary health facilities in the Kakamega County catchment area. Each facility was managed by a PHFM who also had clinical responsibilities. All PHFMs within the county were considered eligible to participate in the cross-sectional study. With a target population of 125 PHFMs, using the most conservative population proportion value of 50%, a 95% confidence level and 0.05 margin of error, 94 participants were required for the study.^27^ We used a simple random technique to select participants for the survey. All PHFMs were listed by name and assigned unique serial numbers from 1 to 125. The ‘sample’ command in R statistical software was subsequently used to generate a set of 94 random numbers between 1 and 125, both inclusive. Facility managers with serial numbers corresponding to the randomly selected numbers were included in the study sample.

Participants for the cross-sectional survey were contacted two weeks ahead of intended interview dates, with follow-up calls to confirm appointment a few days to the interview. Because of the busy schedule of PHFMs, the interviews were scheduled early in the morning before clients arrived or late in the evening after clients had left. One of the researchers (RNC) went to the participants’ offices to administer the survey. The questionnaire was interviewer-administered rather than self-administered, in order to reduce item non-response bias. Data were cleaned and entered into SPSS version 16. Descriptive statistics and cross-tabulations were conducted.

## Results

### Demographic Characteristics of Participants

Of the 94 PHFMs we interviewed, most were female (72%). The most common cadre was credentialed registered nurses (77%), followed by enrolled nurses (18%), and clinical officers (5%). A substantial proportion of participants had cumulative clinical work experience of more than 10 years (43%), although nearly half (44%) of all participants had served at their current facility for less than one year (Table 1).

**Table 1:** Cadre and Years of Clinical Experience of Primary Healthcare Facility Managers in Kakamega County (n = 94)

**Characteristic**	**Frequency**	**Percent**	**95% CIs**
Female	68	72.3	62, 81
Cadre			
Clinical officer	5	5.3	2, 12
Registered nurse	72	76.6	67, 84
Enrolled nurse	17	18.1	11, 27
Years of experience as a health worker			
1-3 years	19	20.2	13, 30
3-5 years	24	25.5	18, 35
5-10 years	10	10.6	6, 19
Over 10 years	41	43.6	34, 53
Length of service at current health facility			
Less than 1 year	44	46.8	37, 57
1-3 years	19	20.2	13, 30
3-5 years	24	25.5	18, 35
5-10 years	5	5.3	2, 12
More than 10 years	2	2.1	1, 8

### Workload and Implementation of Immunization Program

The number of clinical providers typically on duty in the healthcare facilities ranged from one to five, with the median number being two (IQR: 2, 3). Although most respondents (86%) reported seeing at least 40 patients per day, we could not calculate the clinician/patient ratio for healthcare facilities because the patient load information we collected was specifically for the respondent and not the healthcare facility (Table 2).

**Table 2:** Numbers of Client Seen by Facility-in-Charge and Number of Clinical Providers Typically on Duty in Primary Healthcare Facilities in Kakamega County (n = 94)

**Characteristic**	**Frequency**	**Percent**
Number of clients seen by respondent on a typical day		
10-20	6	6.4
21-40	7	7.5
41-60	58	61.7
61-80	8	8.5
>80	15	16.0
Number of clinical providers on duty at the health facility on a typical day		
1	8	8.5
2	46	49.5
3	27	28.6
4	4	4.4
5	9	9.6

Participants generally felt that workload affected implementation of the immunization program (84%). This workload was reported to influence immunization programming by generating longer client wait times that resulted in clients leaving without being vaccinated (45.8%). Inadequate counselling of clients on the importance and schedule of vaccines was another result. The most important contributors to high workload were high patient to staff ratio (68%). PHFMs spending time on non-nursing duties was however the least reported contributor to workload (12.8%) (Table 3). Employment of more clinical staff was the most frequently selected measure to reduce workload (87%), followed by expanding workspace for attending to patients (Table 3).

**Table 3:** PHFMs’ Perspectives on Workload, Effects on Immunizations, and Suggestions for Improvement in Their Facilities in Kakamega County, Western Kenya (n = 94)^a^

**Characteristic**	**Frequency**	**Percent**	** 95% CIs**
Agreed that workload affect immunization programming	79	84.0	77, 91
Perceived effect of workload on immunization program			
Reduced accuracy of reporting	36	38.3	29, 49
Inadequate counselling of clients on the importance of vaccines	36	38.3	29, 49
De-motivated staff	22	23.4	16, 33
Clients waiting for long leading to drop out and missed opportunities	43	45.8	36, 56
Factors contributing to high work load			
High patient/staff ratio	64	68.1	58, 77
Inadequate workspace	22	23.4	16, 33
Staff spending working hours away from facility	19	20.2	13, 30
Multiple registers for facility records	14	14.9	9, 24
Other (eg, high disease burden for malaria, etc)	14	14.9	9, 24
Time spent on non- nursing duties	12	12.8	7, 21
Measures to reduce workload at primary health facilities			
Employing more staff	82	87.2	79, 93
Provide more workspace	26	27.7	19, 38
Provide fridge and gas cylinder for the facility	6	6.4	3, 14
Increase frequency of supervision activities	2	2.1	1, 8
Closer monitoring of work schedule	2	2.1	1, 8

Abbreviation: PHFMs, primary healthcare facility managers. ^a^Answer choices were not mutually exclusive. Respondents were requested to answer yes/no independently for each response option.

### Staff Motivation

Only 13% of participants reported being very motivated to execute their role as a health worker generally, and only 20% reported being very motivated to ensure that all children within their catchment areas were vaccinated. Having vaccination targets for healthcare workers, financial incentives for meeting specified vaccination targets and providing training updates on immunization programming were the most frequently selected measures to improve staff motivation (Table 4).

**Table 4:** Self-assessment of Motivation and Suggestions to Improve Motivation Among PHFMs in Kakamega County, Western Kenya (n = 94)

**Characteristic**	**Frequency**	**Percent**	** 95% CIs**
Self-described motivation level			
Not motivated	39	41.5	32, 52
Motivated	43	45.7	36, 56
Very motivated	12	12.8	7, 21
Self-reported motivation to ensure full immunization coverage			
Not motivated	29	30.9	22, 41
Motivated	46	48.9	39, 59
Very motivated	19	20.2	13, 30
Measures to improve staff motivation^a^			
Financial incentives for meeting specified vaccination targets	48	51.1	41, 61
Training updates on immunization program	42	44.7	35, 55
Improved working conditions and environment	37	39.4	30, 50
Provision of improved storage and cold chain facilities	37	39.4	30, 50
Recognition for meeting specified vaccine target	7	7.5	4, 15
Encouragement on how to meet targets	7	7.5	4, 15

Abbreviation: PHFMs, primary healthcare facility managers. ^a^Answer choices were not mutually exclusive. Respondents were requested to answer yes/no independently for each response option.

### Supervisory Visits

Half of the participants had received a general supervision visit at least twice in the preceding quarter, but only 18% had received any immunization-relevant supervision visit during that same period. Measures perceived to be effective for improving supervision included acting on the feedback from supervisees (26%), improved interaction with facility staff to understand their concerns, and increasing frequency of supervision visits (20%). Strikingly, one-third of participants were reluctant to suggest any measure to improve supervisory visits, because they felt that their prior suggestions had not been taken seriously (Table 5).

**Table 5:** Frequency of Supervisory Visits in the Preceding Quarter and Suggestions to Improve Visits in Primary Healthcare Facilities in Kakamega County, Western Kenya (n = 94)

**Characteristic**	**Frequency**	**Percent**	**95% CIs**
Frequency of general supervision visits in the last 3 months			
None	7	7.5	4, 15
Once	36	38.3	29, 49
Twice	48	51.1	41, 61
Other	3	3.2	1, 10
Frequency of immunization-specific supervision visits in the last 3 months			
None	51	54.3	44, 64
Once	26	27.7	19, 38
Twice	15	16.0	10, 25
Other	2	2.1	1, 8
Endorsed strategies to improve visits in primary healthcare facilities			
Improve implementation of supervisees suggestions	24	25.5	18, 35
Spend more time talking to facility staff during visits	19	20.2	13, 30
Increase the frequency of supervision visits	19	20.2	13, 30
Other; Nothing (nothing has ever changed)	32	34.0	20, 35

## Discussion

In this study, we explored how PHFMs in western Kenya regarded how human resource factors influenced program effectiveness in their facilities. We found that managers largely perceived themselves to be overworked and unmotivated with low supportive supervision, both generally and specifically in terms of immunization. While these findings align with those that have been reported in prior studies about health workers generally across sub-Saharan Africa,^22,28^ to our knowledge this is the first quantitative report of PHFM perspectives on how human resource factors influence programs in primary healthcare facilities in Kenya.

Our findings indicate that almost half of PHFMs had spent a year or less in their current duty posts. This might reflect frequent turnover of facility leadership and clinical staff- a human resource factor that can influence performance. Another potential explanation was that the timing of our survey was just close to a period when widespread reshuffling of staff across healthcare facilities had occurred. Unfortunately, neither the cross-sectional survey nor the preceding interviews probed this issue.

Missed opportunities stemming from the potentially modifiable issues observed in our study (eg, inadequate counselling, poor record-keeping, long patient wait times, high workload) is particularly frustrating considering the challenges involved in having both patients and health technologies present at the facility in low resource settings. Patients often have to overcome an array of challenges to present for care at a primary healthcare facility.^29^ Furthermore, significant attention has been paid in the last decade to innovations for maintaining stock of health technologies and improving supply chain logistics in primary care facilities in Kenya and elsewhere, to ensure vaccines and other health technologies are available in good condition at the last mile of delivery.^30,31^

This research adds to the literature on the patient experience before and during their interactions with primary healthcare providers in sub-Saharan Africa. Appropriate counselling and interaction with clients are key components of effective programs.^32^ Effective counselling can improve awareness and shape community norms regarding vaccination and other primary care services, while lack of adequate information can limit community demand and lead to failure to return for subsequent healthcare visits.^33^ Furthermore, experiencing long wait times and poor counselling during prior visits, dissuades patients from returning for follow up.^34^

Inadequate staff motivation plausibly influences program effectiveness. Motivation is a complex construct that interacts with many other factors of the workplace environment.^35,36^ Traditionally, it has been viewed as two dimensional, with internally generated (intrinsic) sources and externally generated (extrinsic) sources. For example, the motivation to vaccinate all children would be considered intrinsic if driven by desire to help the community but extrinsic if driven by desire to reach workplace goals.^37^ Recent scholarship has called for consideration of the multidimensional nature of motivation, inclusive of multiple origins, sources, and regulatory mechanisms,^38^ so that motivation to vaccinate all children would be better characterized as a complex mix of internal and external sources of regulation.

Given our approach of measuring motivation with direct questions, this study had a limited characterization of motivation. However, considering potential for social desirability biases, we suggest that the small proportion of PHFMs self-rating themselves as highly motivated is likely to be accurate. This low level of motivation and commitment to job duties may interact with the heavy workload to translate into frequent missed opportunities in primary healthcare delivery.

The lack of immunization-relevant supervisory visits within the preceding 3 months might indicate that supervisory exercises were being observed as a perfunctory audit activity. Supervision should not be a periodic exercise but an ongoing process for effective program delivery.^39^ Prior research has shown that supportive supervision, defined as workers feeling valued, motivated, and guided by an accessible supervisor is associated with increased program delivery indicators, but operational or structural supervision is not.^40^ In our study, many participants were reluctant to provide suggestions on how to improve supportive supervision because they considered prior suggestions not to have been implemented. Unfortunately, due to the close-ended nature of the survey it was not clear whether this reflected a widespread perception of not being taken seriously by the district-level managers. A recent systematic review showed that supportive supervision in sub-Saharan Africa may be most effective when focused on problem-solving.^41^ Therefore, a strategy to engage supervisors in addressing obstacles, may yield improved outcomes.

Furthermore, if PHFMs are not engaged in models of supportive supervision, it is likely that they are not providing supportive supervision to the clinicians that they manage and work with. This propagates the observed missed opportunities due to modifiable factors. A systematic literature review of randomized control trials of interventions to improve health worker performance in sub-Saharan Africa identified inadequate supervision and management and lack of follow-up support as most frequent modifiers of intervention success.^42^ Feedback sessions with clinical officers in Kenya improved adherence to clinical quality guidelines—while supporting social cohesion, pride in work, and self-esteem.^43^ These workplace environment and personal factors are closely linked with health worker motivation,^9,35^ another glaring gap in our study context to achieving high vaccination coverage.

Addressing workplace factors such as supervision and workload are some of the multiple components that can be levered to improve self-reported low motivation. After employing more staff, offering financial incentives contingent on immunization outcomes was the most frequently endorsed strategy. In recognition of the multidimensional nature of motivation,^38^ and the literature evaluating different types of incentives,^44^ we propose that financial incentives should be only one component of a comprehensive effort. In this context, increasing the frequency and changing the nature of supervisory visits so that PFHM feedback is acted upon, are potential strategies to improve morale and help timely resolution of program bottlenecks.

## Strengths and Limitations

The strengths of this paper include highlighting the perspectives of an important cadre in the primary healthcare system, whose voice has been under-represented in the literature on interventions to strengthen primary healthcare programs. In addition, our survey was based on formative research done in the same setting; hence our findings are likely to have high contextual validity. Also, respondents were randomly sampled, thus being representative of PHFMs in the county.

However, our findings should be generalized beyond this population with caution. Participants for this study were drawn from primary healthcare facilities in a single county in western Kenya. Given the likelihood of contextual differences in operational constraints, socio-economic conditions, and political economy of the primary healthcare system across different settings, further studies are needed to define the inferential boundaries and relevant contexts in which our findings can be applicable, beyond the immediate context. However, recent findings of low motivation and poor supervision affecting immunization programming in Nigeria suggest that our findings might be relevant in other African settings.^22^ In addition, the focus of this paper is on government policy and public facilities. There are faith-based organizations and private healthcare facilities with little government oversight and management structure in the county. It is likely that staffing patterns and perceptions of managers in these facilities might differ from those reported in this paper in important ways.

Furthermore, this study elicited perspectives of PHFMs because of their unique position in the impact pathway of primary healthcare programs, including routine immunization. Evaluation data showing that, in fact, accuracy of reporting or other clinical quality measures are associated with workload and other perceived problems would be a more objective indication of the importance of the perceived problems. While we argue that the role of the respondents puts them in a position to have unique insights into contextually relevant intervention targets, perception is of limited utility unless it correlates with actual performance and functional outcomes. Moreover, the classical concerns about social desirability and validity of single item questions in eliciting accurate perspectives about complex constructs like motivation and supervision, limit confidence in our findings. Future work should use validated scales to shed light on the nuances of the multidimensional nature of these constructs among PHFMs.^38^

## Conclusion

PHFMs occupy a unique position in the primary healthcare system, where their perspectives often shape program delivery. Their views about appropriate solutions to operational bottlenecks and human resources issues such as workload, supervision, and staff motivation can provide contextually relevant information about potentially effective interventions for strengthening primary healthcare delivery. Besides increasing clinical staff, introducing some financial incentives contingent on specified targets, and making supervisory visits meaningful with action on feedback, were endorsed by PHFMs as strategies to increase program effectiveness in primary healthcare facilities in Kenya. Further studies are needed to evaluate the importance of incorporating perspectives of PHFMs in developing comprehensive solutions for improving primary healthcare programs in Kenya.

## Acknowledgements

We thank the PHFMs and members of the district and county health management team in Kakamega County, Kenya for participating and sharing their time and insights with us. We appreciate the contributions of Stephanie Martin and staff at the Kakamega regional vaccine depot to the data collection instruments.

## Ethical issues

Ethical clearance for this research project was obtained from the AMREF Ethics Review committee and written permission to work in the county health system was granted by the Kakamega county chief officer of health. A research permit was obtained from the Kenyan National Commission for Science, Technology and Innovation (NACOSTI). Written informed consent was obtained from all study participants.

## Competing interests

Authors declare that they have no competing interests.

## Authors’ contributions

Conceptualized study: MOO and RNC; Study Design: MOO, RNC, SO; Data analysis and interpretation: MOO, RNC, SO, RCS; Manuscript draft: MOO and RNC; Revised draft for intellectual content: RNC, RCS, SO, MOO.

## Authors’ affiliations

^1^University of Nairobi, Nairobi, Kenya. ^2^Center for Global Health, Arizona State University, Tempe, AZ, USA. ^3^State University of New York at Buffalo, Buffalo, NY, USA. ^4^Massachusetts General Hospital, Boston, MA, USA. ^5^Harvard Medical School, Boston, MA, USA.

## Key messages

**Implications for policy makers**

- This research reinforces the need for policy makers to investigate perspectives of primary healthcare facility managers (PHFMs) in developing appropriate solutions to strengthening primary healthcare delivery.
- For policy-makers in Kenya and similar settings, this research highlights the importance of improving the way supervisory activities are conducted.
- Our findings emphasize healthcare worker motivation as a key factor that can be improved to strengthen primary healthcare delivery.

**Implications for public**

Although this research focuses on primary healthcare facility managers (PHFMs), and not directly on their clients, the findings have indirect implications for their clients. PHFMs occupy a unique position in the primary healthcare system, where their perspectives, capacities and attitudes often shape how the public experience healthcare. Their perspectives can provide contextually relevant information to develop interventions for strengthening primary healthcare delivery, and improve how the public experiences care delivery. This study identified improvement of supervision and motivation of healthcare staff as an intervention endorsed by PHFM to improve care delivery.
